# Targeting the Gut Mucosal Immune System Using Nanomaterials

**DOI:** 10.3390/pharmaceutics13111755

**Published:** 2021-10-21

**Authors:** Jacob McCright, Ann Ramirez, Mayowa Amosu, Arnav Sinha, Amanda Bogseth, Katharina Maisel

**Affiliations:** Fischell Department of Bioengineering, University of Maryland, 8278 Paint Branch Drive, College Park, MD 20742, USA; mccright@umd.edu (J.M.); adramire@umd.edu (A.R.); mayowa@umd.edu (M.A.); asinha27@umd.edu (A.S.); abogseth@umd.edu (A.B.)

**Keywords:** gastrointestinal tract, lymph node, gut-associated lymphoid tissues, immunotherapy, vaccine, lectins, microfold (M) cells

## Abstract

The gastrointestinal (GI) tract is one the biggest mucosal surface in the body and one of the primary targets for the delivery of therapeutics, including immunotherapies. GI diseases, including, e.g., inflammatory bowel disease and intestinal infections such as cholera, pose a significant public health burden and are on the rise. Many of these diseases involve inflammatory processes that can be targeted by immune modulatory therapeutics. However, nonspecific targeting of inflammation systemically can lead to significant side effects. This can be avoided by locally targeting therapeutics to the GI tract and its mucosal immune system. In this review, we discuss nanomaterial-based strategies targeting the GI mucosal immune system, including gut-associated lymphoid tissues, tissue resident immune cells, as well as GI lymph nodes, to modulate GI inflammation and disease outcomes, as well as take advantage of some of the primary mechanisms of GI immunity such as oral tolerance.

## 1. Introduction

The gastrointestinal (GI) tract is the largest mucosal surface of the body, with >400 m^2^ of surface area facing the external environment. Due to its constant exposure to external stimuli and microbes, the gut has evolved with an extensive association of immune tissues, including Peyer’s patches and lymph nodes that are responsible for keeping harmful materials out of the body’s internal environment. Due to its large absorptive capacity, the gut has been the primary target for delivering drugs for systemic and local treatments. In recent years, with the increasing popularity of immune modulatory treatments, the gut immune system has become a target for modulating immunity for the treatment of local gut inflammatory conditions and beyond. This can be leveraged using nanoparticles and nanomaterials optimized for mucosal delivery. Nanoparticles and nanomaterials can be engineered to effectively interface with and cross key barriers within the GI, as well as be engineered to reach key immune effector sites. In this review, we provide an overview of gut anatomy and immunity, followed by a description of nanomaterial-based therapeutic systems that target different components of gut immunity, including the gut-associated lymphoid tissues, lymph nodes, immune cells, and oral tolerance mechanisms.

## 2. Overview of Gut Anatomy

### 2.1. Mucus and Epithelium

Mucus is the first barrier that protects mucosal surfaces from harmful pathogens and particulates [1]. Mucus effectively traps pathogens and particulates due to its chemical and physical properties. It is a semipermeable hydrogel that is composed of mucin fibers, long peptides coated with glycans (peptidoglycans) possessing an overall negative charge and ranging in size from 200 kDa to 200 MDa [2,3]. The overall negative charge of mucins allows for electrostatic interactions with positively charged pathogens or particulates and thus traps them. Further, the mucin fibers have hydrophobic regions that interact to form bundles that can trap hydrophobic materials [4]. Mucin fibers also link with each other leading to a mesh-like structure, thus allowing exclusion based on size of particulates. Mucus composition can vary significantly in structure and thickness at different mucosal surfaces and in different sections of the GI tract [4]. Crossing the mucus barrier poses a significant hurdle for delivering therapeutics to the mucosal surfaces, including the GI tract, and has been the focus of several other excellent reviews [4,5].

The mucosal epithelium is the second barrier that must be crossed for effective drug delivery to mucosal surfaces, and its permeability is highly regulated [4]. In the gut, the mucosal epithelium is largely made up of enterocytes and goblet cells (mucus-producing cells). The enterocytes are responsible for nutrient absorption and form a tight barrier against pathogens and particulates via tight and cell–cell junctions. These tight and cell–cell junctions specifically mediate paracellular transport in the gut and thus act as the main barrier to materials. Transmembrane proteins known as claudins play a key role in the barrier properties of tight and cell–cell junctions and have been investigated extensively as a target to enhance drug delivery at the epithelium [4,6]. The gut epithelium can respond to changes within its microenvironment and trigger immune responses by secreting immune modulatory molecules like cytokines. Gut immunity is further enabled through the presence of immune cells in the epithelium and basement membrane, including CD4+ and CD8+ T cells, B cells, dendritic cells, and macrophages [4]. In addition, Paneth cells can produce antimicrobial peptides and neuroendocrine cells can detect harmful substances within the gut [7]. The gut also contains secondary lymphoid structures known as gut-associated lymphoid tissues (GALT) that perform local immune functions (see GALT section below). These features allow the mucosal epithelium to serve as both a cellular and physical defense system against harmful pathogens and materials encountered in the gut.

### 2.2. Lymphatics

The lymphatic system is an essential contributor to homeostasis within the body, including the gut [8]. This system largely consists of lymphatic vessels and capillaries, which are pervasive throughout the body. These vessels function to collect and transport lymph from tissues via a set of vessels and across a system of lymph nodes back into systemic circulation [9]. Lymph is a liquid formed from the interstitial fluid surrounding tissues and contains immune cells, extracellular proteins, antigens, and excess plasma from blood [10]. Lymphatic capillaries are permeable to macromolecules and obtain the lymph through osmotic pressure from the surrounding tissues. Lymphatic vessels are the main source of propulsion of lymph towards the lymph nodes and ducts, which take the fluid to the subclavian vein and back to the heart. In the gut, lymphatics have specialized capillaries, known as lacteals, that transport dietary lipids along with immune cells and other molecules absorbed from the interstitial fluid from the intestine [11]. Consequently, the blockage of lymph uptake within the intestines can lead to the development of obesity and other metabolic issues [12]. Intestinal inflammation can also lead to the dysregulation of lymphatic transport, which may be one of the underlying causes of chronic inflammatory conditions such as inflammatory bowel disease [13]. Overall, the efficient transport of lymph from the interstitium to systemic circulation via the lymphatic vessels and their downstream lymph nodes is essential for maintenance of homeostasis and these transport functions can be harnessed for therapeutic drug delivery.

### 2.3. Interstitium

Interstitial tissue spaces surround all cells within our organs and connective tissues, and are the primary source of lymph fluid. Recent efforts have demonstrated that the interstitial tissue consists of extracellular matrix, usually collagen, bundles that surround fluid-filled spaces and cells lining these bundles on one side [14,15]. The space forms an interconnected network, similar to a hydrogel, with largely unknown pore sizes that likely vary between different tissues. In the gut, researchers have shown that digested materials enter the interstitial tissue after being transported across the mucosal epithelium and from there they are distributed either into lymphatic or blood capillaries for systemic delivery. Extracellular matrix proteins also contain charged and targeting moieties that can aid in cell migration and motility [15]. Due to its porous structure and potential adhesion to the extracellular matrix or interstitial cells, therapeutic delivery through the interstitium poses a challenge, particularly when trying to reach immune cells embedded in the interstitium or lymphoid structures lying beneath it.

## 3. Overview of Gut Immunity

### 3.1. Gut-Associated Lymphoid Tissues (GALT)

**Figure 1:**
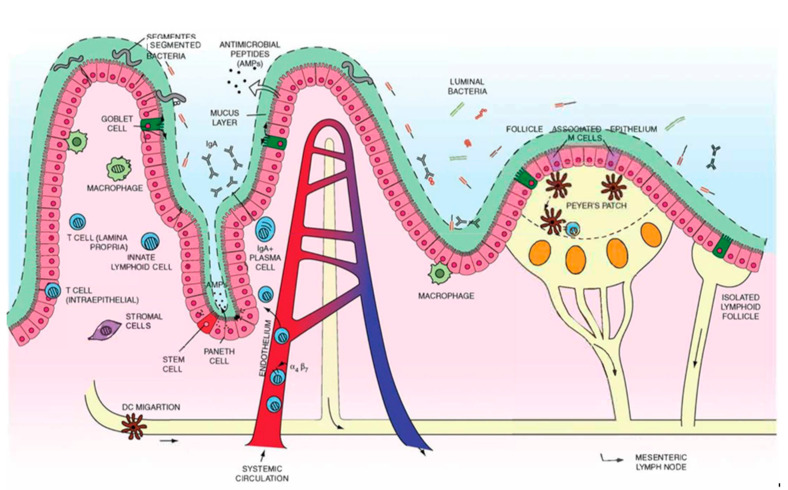
Summary of mucosal immune system components including isolated lymphoid follicles, lymphatic vessels, Peyers patches, mesenteric lymph nodes, and immune cells within and surrounding the epithelium. Reproduced with permission from [20]. Copyright BMJ Publishing Group Ltd., 2013.

The gastrointestinal tract is a key interface between the outside world and the rest of our bodies, and as such, contains a vast diversity and number of immune cells and immune-relevant sites. These sites play an important role in forming and modulating local immunity. As a result, topics like oral vaccine development and compartments within the GI tract that affect immunity have been subjects of intense study for many years. Within the GI tract, the immune response is formed in both the gut-associated lymphoid tissue (GALT) and the mesenteric lymph nodes (mLNs) and these have been of particular interest for vaccine delivery and understanding of how to influence inherent immune mechanisms such as oral tolerance [16]. The GALT consists of two main classifications of tissues: multi-follicular lymphoid tissues including Peyer’s patches, and isolated lymphoid follicles distributed along both the small and large intestines (Figure 1) [17,18,19,20]. 

#### 3.1.1. Multi-Follicular Lymphoid Tissues

Peyer’s patches are the primary multi-follicular lymphoid tissue. They are key sites where adaptive immunity is formed and contain microanatomical niches for effective immune priming and propagation [17]. There are up to hundreds of Peyer’s patches found on the antimesenteric wall of the small intestine, with increasing density toward the terminal ileum, where they form a ring at the ileocecal junction between the small and large intestine [21]. Specialized follicle-associated epithelium, populated with microfold cells (M cells) and intraepithelial lymphocytes under a sparse covering of mucus, are found on the luminal side of a Peyer’s patch [22]. M cells cover many GALT beyond Peyer’s patches and serve to actively transport luminal antigens via transcytosis into the parenchyma to generate IgA-mediated adaptive immune responses. M cells also express a large number of glycosylated moieties on the surface that are thought to interact with the intestinal microbiome [16]. On the basolateral side, M cells interact with immune cells within the Peyer’s patch. Underneath the epithelium, the follicular and interfollicular areas contain a germinal center with proliferating B cells and antigen-presenting cells, including dendritic cells and macrophages. Between the central follicle lies the subepithelial dome where a mix of cells including B cells, T cells, macrophages, and dendritic cells reside. Peyer’s patches contain their own vasculature, where naïve lymphocytes migrate to efferent lymphatic vessels from the mesenteric lymph nodes [17,23,24].

#### 3.1.2. Isolated Lymphoid Follicles

Isolated lymphoid follicles (ILFs) are single lymphoid follicles, making them much smaller than Peyer’s patches, and constitute a major component of the GALT. ILFs, like Peyer’s patches, contain a follicle-associated epithelium rich in M cells that shuttle antigen from the lumen into the parenchyma. Dendritic cells, macrophages, T cells, and B cells reside under the follicle-associated epithelium shaping the GI tract’s adaptive immunity [25,26,27]. Unlike Peyer’s patches, an ILF does not contain plasma cells (mature B cells that produce antibodies), and contains high proportions of naïve and memory B cells [28].

### 3.2. Lymph Nodes

The mesenteric lymph node (MLN) is one of the first key sites where nutrients and microbial substances enter the lymph from the gut through the lamina propria. Lymph nodes are key mediators of adaptive immunity and are made up by the cortex, paracortex, and medulla. Within these regions are housed massive numbers of lymphocytes and antigen presenting cells. The cortex houses B cells and can be found on the outer edges of the lymph node. Towards the center of the node, the medulla houses T cells along with dendritic cells and other antigen-presenting cells [29,30]. Materials enter lymph nodes via blood or lymphatic vessels, from systemic circulation or peripheral tissues, respectively. Lymphatics transport most antigens from the gut to the MLN either as a soluble form or via antigen-presenting cells. T cells in the MLN are then educated to form adaptive immune responses against antigens and pathogens [31].

### 3.3. Lymphatic Endothelial Cells Transport Antigens and Modulate Immunity

Lymphatic vessels exist both within lymph nodes and the lacteals within the villi of the gut [32]. Lymphatic endothelial cells (LECs), which make up lymphatic vessels, stem from venous progenitor cells, however, they have distinct lymphatic markers: vascular endothelial growth factor receptor 3 (VEGFR-3), the prospero homeobox-1 fate determining transcription factor (Prox-1), lymphatic vascular endothelial hyaluronan (LYVE-)1, and podoplanin [33,34]. In addition to forming the vessels that transport fluid from peripheral tissues (i.e., lamina propria within the gut), LECs also have a key role in regulating both adaptive and innate immune responses. LECs secrete chemokines that recruit immune cells to the lymph nodes, including CCL21, which is responsible for the recruitment of dendritic cells and naïve T cells [34]. LECs also modulate the immune response through expression of PD-L1, which can lead to dysfunctional activation of T cells when interacting with LECs via MHCII [35,36,37]. LECs express MHCII and can acquire MHCII-antigen complexes from other antigen presenting cells, such as dendritic cells [37].

### 3.4. Oral Tolerance

Oral tolerance refers to the body’s attenuated response to antigens from food and microbes within the gut. This ensures that we do not unnecessarily respond to non-harmful molecules and prevents killing of our commensal microbiota. Oral tolerance is mediated through the active suppression of immune responses to antigens first encountered in the gut. Dysregulation of oral tolerance is thought to be partially responsible for inflammatory conditions such as food allergies and inflammatory bowel disease [38,39].

In oral tolerance, antigens are transported from the lumen of the gut across epithelial cells, eventually reaching lymphoid tissues and lymphocytes within the lamina propria via lymphatic vessels or migratory antigen-presenting cells, such as CD103+ dendritic cells. After capturing antigens, CD103+ dendritic cells migrate from the intestine to the mesenteric lymph nodes, where they induce regulatory CD4+ T cells via TGFβ and retinoic acid. Therefore, oral tolerance is mediated by CD4+Foxp3+ regulatory T cells that are peripherally induced rather than thymically developed. Once regulatory T cells are induced, they migrate from the lymph node to the lamina propria in the gut in a CCR9-mediated mechanism. In the lamina propria, CX3CR1^+^ macrophages produce IL-10, which is critical in the expansion of Tregs and overall proper induction of oral tolerance [38,40,41]. Additional mechanisms of oral tolerance are T cell anergy and T cell depletion. Anergy occurs when high levels of antigen are present in the GI tract, and yields T cells that are unresponsiveness to the antigen [42]. Depletion occurs when antigen specific T cells undergo apoptosis, which can be induced through the interaction with regulatory T cells.

Tolerance is induced within the MLN and while Peyer’s patches, ILF, and liver tolerance mechanisms may also have a role in oral tolerance, these are not required for the induction of oral tolerance. Interestingly, recent work has demonstrated that specifically the MLN-draining antigens from the early gut, including duodenum and early jejunum are responsible for tolerance induction. Researchers showed that removal of these lymph nodes resulted in a lack of tolerance to a variety of oral antigens and microbes in mice [43]. Targeting these earlier lymph nodes may thus be a crucial step toward taking advantage of oral tolerance for therapeutic purposes.

## 4. Nanomaterials for Targeting the Gut Mucosal Immune System

Nanomaterials (NMs) are materials, organic or inorganic, generally in the range of 1–500 nm in size. They are produced using a variety of methods including, but not limited to, nanoemulsions, nanoprecipitation, printing, and jetting. They have gained popularity over the years not only due to their size, but also to their customizability with, e.g., targeting molecules, potential for loading and protecting cargo, and interaction with different biological barriers. The specific size, shape, reactivity, and other characteristics are altered appropriately for the application in question (Table 1). Nanomaterials have received considerable attention for targeting therapeutics to the GI tract, since a shell of polymer or other materials can shield precious drug cargo from digestion in the harsh environments of the gut. Nanomaterials have also been shown to effectively target certain immune cells that are more likely to endocytose or phagocytose larger materials over small molecules or proteins. As such, nanomaterials are ideal for targeting the gut mucosal immune system [4]. Below, we describe how different components of the gut immune system, including GALT, lymph nodes, and specific immune cells, have been targeted using different nanomaterial strategies.

### 4.1. GALT Targeting

To modulate gut immunity, the gut-associated lymphoid tissues, or GALT, are a natural target. GALT help shape local immune responses and have lymph-node-like features that allow for the development of not only innate but also adaptive immune responses. The primary sampling of luminal contents by M cells presents an opportunity to take advantage of existing trafficking mechanisms that can deliver therapeutics to the GALT immune cells. Direct delivery of immunomodulatory treatments to GALT potentiates immunotherapies. Targeting these structures has been achieved in a variety of ways, including through the use of sub 500 nm nanomaterials that often have surface modifications, such as targeting ligands that bind M cells that form the luminal cell layer on top of the secondary lymphoid tissues (Table 2). Studies demonstrated that thiol-organosilica nanoparticles smaller than ~700 nm were more frequently found in Peyer’s patch or other M-cell-rich regions [70]. The authors specifically tested 95, 110, 130, 200, and 340 nm nanoparticles and demonstrated that the fluorescence area covered by these sizes was significantly more than that of 695 and 1050 nm. Using immunofluorescence, they also found that these smaller nanoparticles colocalized with M cells and CD11b+ cells, including macrophages and dendritic cells, indicating that smaller sizes are preferable for M cell targeting. The authors also demonstrated that both transcellular and paracellular transport pathways were involved in uptake and distribution of the nanoparticles in the GALT regions. Many studies since have used nanoparticle systems ranging 50–500 nm in size, well within the optimal size range for reaching GALT.

Several studies have utilized mucoadhesion to enhance M cell uptake of nanomaterials. M cells regions are not rich in mucus-producing cells, and thus are coated in a thinner layer of mucus. Nanomaterials that stick to the mucus layer are thus likely to be picked up by M cells and transported across to the underlying secondary lymphoid structures. Mucus contains mucin proteoglycans, protein chains that have hydrophobic domains and highly negatively charged glycosylations, which effectively trap hydrophobic materials, such as lipids, as well as positively charged materials, such as chitosan. Bachhav and colleagues reported that a lipid─polymer hybrid nanoparticle (termed LIPOMER) was able to effectively enhance sticking of 300–400 nm nanoparticles to the Peyer’s patches, using glyceryl monostearate as primary lipid [71,72]. The group reported finding that nanoparticles were highly associated with Peyer’s patches and had low accumulation in the liver compared to non-lipid-coated polymeric nanoparticles, suggesting that LIPOMERS were able to reach systemic circulation via lymphatic vessels. They followed up on this study, testing if a non-lipid hydrophobic polymer, ethyl cellulose, could also function to enhance mucoadhesion and thus enhance GALT targeting. The group found that their GantrezAN-110 nanoparticle formulation was also able to enhance Peyer’s patch uptake and reduce liver concentration of their model drug rifampicin, suggesting that nanoparticles were transported via lymphatic vessels away from the GALT. Additionally, several groups have reported using chitosan to enhance nanoparticle uptake by GALT. Kadiyala et al. demonstrated that DNA-loaded chitosan nanoparticles were effectively transported in vitro, with M cell cultures having 5-fold higher transport than enteroid-like cells, and additionally showed that transferrin enhanced this transport in both models [73]. They hypothesized that soluble chitosan in a low pH environment can serve as a permeation enhancer, which may in part be responsible for the increased paracellular transport of their DNA nanoparticle system. More recently, Shim et al. utilized chitosan nanoparticles to target brucellosis, a worldwide zoonotic disease that affects both humans and domestic animals. The group had previously found that loading chitosan nanoparticles with *Brucella abortus* antigen, malate dehydrogenase (Mdh) (which has been shown to elicit partial protection and immunostimulatory effects against brucellosis), elicited systemic IgA responses in vivo and proinflammatory cytokine production in ThP-1 cells. In this study, the authors showed that chitosan nanoparticles increased Mdh transport across and IL-1β and IL-6 production by M cells in vitro. Additionally, they found evidence that MyD88-dependent signaling through the toll-like receptor 2 was activated by the Mdh chitosan nanoparticle system, suggesting that Mdh and the nanoparticle system synergistically enhance the type 2 immune response elicited that may contribute to protection against brucellosis. Altogether, these and other studies have shown that mucoadhesive nanomaterials can enhance uptake of antigens and other therapeutics by M cells, modulating immunity and enhancing systemic drug delivery.

Research has also focused on specifically targeting molecules expressed by M cells to maximize uptake and delivery of nanomaterials to GALT. Early work identified peptide sequences through phage display [74] that adhere specifically to M cells via, e.g., sugar residues, such as α-l-fucose, specifically expressed by M cells [75,76]. One of the most ubiquitously used targeting moieties are lectins, such as the Ulex europaeus agglutinin 1 (UEA-1), a lectin that binds to α-l-fucose residues found on the apical side of M cells. Many studies have used this ligand and we refer readers to these excellent reviews for earlier work [75,76]. More recently, lectins and peptides have been used to target immune modulatory therapeutics to the GALT. For example, Du et al. reported a PLGA nanoparticle system containing a DNA vaccine or protein targeted to M cells using UEA-1 that could increase IgA levels in mice and piglets [77]. They demonstrated that nanoparticles without targeting enhanced IgA levels, but that UEA-1 addition further enhanced both IgG and IgA levels in animals receiving the DNA vaccine, indicating that utilizing an M cell targeting strategy may enhance mucosal vaccine efficacies. Additionally, Malik et al. used UEA-1 to target alginate nanoparticles containing the model antigen albumin to M cells and demonstrated that their vaccine enhanced serum IgG1 and IgG2a, as well as mucosal IgA levels compared to traditional alum-based vaccines [78]. Additionally, Lee et al. demonstrated that a new peptide, β-glucan and glycine-arginine-glycine-aspartic acid-serine (GRGDS), can be used to form 200–250 nm nanoparticles when added to the anionic influenza (PR8) antigen via electrostatic interactions [79,80]. They demonstrated that 21 days after immunization, more anti-flu antibodies can be found in serum, intestine, and gut mucus compared to free influenza antigen solution. Shima et al. demonstrated that using an anti-GP2 antibody, which targets glycoprotein 2, one of the antigen uptake receptors of M cells, effectively enhances the immune response induced by oral vaccination against ovalbumin (as model antigen) and *Salmonella typhimurium* [80]. They demonstrated that anti-Gp2 antibodies reduced overall infection by virulent S. Typhimurium compared to lysate alone in mice. Finally, Jian et al. showed that coating nanoparticles with chitosan and CSK9-targeting peptides could enhance oral-vaccine-induced immunity against *Brachyspira hyodysenteriae*. They loaded the membrane protein B of *Brachyspira hyodysenteriae* (BmpB) into nanoparticles as a model antigen, and coated nanoparticles with chitosan and CSK9 [81]. They found that their vaccine enhanced IgA levels in feces and intestine, and IgG1 and IgG2a antibodies in serum against BmpB 21 days after oral administration compared to a free protein solution, as well as protein loaded into nonmodified PLGA nanoparticles and PLGA nanoparticles coated in only chitosan, suggesting that CSK9 targeting most effectively enhanced the response. Altogether, these data demonstrate that targeting M cells and the underlying GALT has the potential to enhance therapeutics targeting the mucosal immune response, which can have significant implications particularly for oral vaccine strategies. We direct readers to an excellent review on M cell-targeting vaccines for more detail [82].

### 4.2. Lymph Node and Lymphatic Targeting

Lymphatics are the conduit from peripheral tissue to the lymph nodes and have received considerable attention as a natural delivery mechanism of immunotherapies and vaccines to the lymph nodes. Therapeutics transported via lymphatics in the gut additionally avoid hepatic first pass metabolism and thus have higher bioavailability. Gut lymphatics can be particularly targeted through lipid-based mechanisms, as the gut lymphatics are responsible for the transport of dietary lipids into systemic circulation. However, there are a few challenges that inhibit the passage of particles into lymphatics and lymph nodes. Initial lymphatics surround the tissue and help collect fluids and foreign particles. These initial lymphatics only allow molecules 10–250 nm in radius to pass through. Materials that are larger than this will get trapped in the extracellular matrix and will be unable to pass and be transported into lymphatic vessels [83]. Here, we describe lipid-based nanoparticle systems that take advantage of dietary lipid pathways, as well as non-lipid-based systems that have been designed to enter gut lymphatics and transport materials to the lymph nodes and beyond.

#### 4.2.1. Lipid-Based Delivery Systems

Dietary lipids are transported by lymphatic and not blood vessels from the gut into systemic circulation. These lipids are packaged into chylomicrons by enterocytes in the gut [84,85] that are exocytosed into the lamina propria and then taken up by lymphatic vessels [84,85]. Targeting the chylomicron pathway leads drugs to be delivered effectively to the local lymph nodes, which can be beneficial for immune modulatory therapies. To take advantage of this process, therapeutics can be made into prodrugs, or lipid formulations (LF), that contain a cleavable lipid component, so they can be packaged into chylomicrons and transported across the gut and into lymphatic vessels via the naturally occurring chylomicron transport mechanisms (Table 1) [86,87,88]. A recent study by Lee et al. explored how to increase systemic exposure and improve oral bioavailability of a highly lipophilic drug, Orlistat [84]. Three LFs were tested and individually emulsified with Orlistat, alongside a lipid-free control: medium-chain fatty acid (MC-FA), long-chain fatty acid (LC-FA), and long-chain triglyceride (LC-TG) [84]. They found that when administered with LC-FA formulations, the cumulative lymphatic transport of Orlistat and TGs was significantly higher than when administered with MC-FA and LC-TG [84]. The peak concentration of the drug in the lymph was found to be around 2–3 h after administration [84]. When comparing this peak concentration across all formulas, LC-FA had the highest concentration [84]. They also found that increasing the dose of LC-FA while keeping the drug dose constant significantly increased lymphatic transport of the drug [84]. However, the increase in LC-FA did not affect TG transport [84]. This study largely took advantage of chylomicron formation by delivering molecules like FAs that can be more easily resynthesized to TGs and assemble into chylomicrons. In another study, mycophenolic acid (MPA), an immunosuppressant, was linked to a TG (MPA-TG) [89]. The researchers aimed to target mesenteric lymphatic vessels and lymph nodes, using the chylomicron pathways, and linked MPA to the 2-position of a diglyceride [89]. They found that there was a higher number of MPA-related molecules found in lymph after intraduodenal administration using MPA-TG compared to just MPA and MPA co-delivered with TG [89]. When looking directly at the MLNs, the group discovered that there was a 20-fold higher concentration in MLNs with MPA-TG compared to MPA alone [89].

Drugs chemically conjugated to a lipid still face potential degradation in the presence of a harsh digestive environment in the gut. To avoid this, researchers have used nanomaterials containing their therapeutic of interest, thus shielding them from digestion, and coated these with lipids to promote integration into chylomicrons. These nanomaterials can be packaged into chylomicrons and show an increase in transport through enterocytes compared to free drug or uncoated nanomaterials. Yin et al. used a lipid-coated nanoparticle formulation to deliver an immunomodulatory drug, Laquinimod (LAQ), to treat Crohn’s disease, an autoimmune disease [90]. They used a mesoporous silica nanoparticles coated with α-α′dilaurin to mimic TGs [90]. They also added an acid resistant coating to protect the nanoparticles from gastric fluids that would otherwise lead to their degradation [90]. When the nanoparticle system was delivered orally to mice with Crohn’s disease, they found that nanoparticles were transported to the lacteals and the downstream mesenteric lymphatic vessels. The group also explored how their drug delivery system affected lymphangiogenesis, which is commonly associated with Crohn’s disease and is thought to be a way to compensate for dysfunctional mesenteric lymphatic vessels [90]. Lymphangiogenesis is mediated by the binding of growth factors VEGF-C and VEGF-D to VEGFR3, and the researchers found that their formulation caused a significant decrease in VEGF-C and VEGFR3 expression compared to control groups. Additionally, their treatment reduced expression of proinflammatory cytokines, suggesting an amelioration of Crohn’s disease overall.

Another way that researchers have taken advantage of the chylomicron pathways is by using solid lipid nanoparticles (SLN). SLNs can be loaded with drugs while simultaneously protecting the load from enzymatic degradation in the gut and acting as lipids in the chylomicron pathway. One group hypothesized that incorporation of insulin in SLNs can promote intestinal uptake and transport into the lymphatics [91]. Their imaging studies demonstrated that insulin-loaded particles were found in the villi, indicating successful transport across the mucosal epithelium [91]. Authors found a significant concentration of insulin (2.0 μg/mL mg^−1^) in the lymph 1.5 h after injecting their formulation into the duodenum of mice [91]. Obinu et al. sought to deliver Genistein via chylomicron pathways [92]. Genistein is used to treat and prevent tumors, cardiovascular diseases, osteoporosis, and hormonal pathologies. To test chylomicron formation in vitro, unloaded SLNs were mixed with phospholipids and cholesterol and added to human enterocyte-like Caco-2 cells capable of forming chylomicrons [92]. They found that after transport across Caco-2 cells, the size of the SLNs was increased from 270 to 390 nm, indicating that they were formed into chylomicrons [92]. These data highlight that SLNs are readily taken up by enterocytes and can be formatted into chylomicrons that are then secreted for transport into the lymphatics.

Lipid-based nanoparticles have also been used to target intestinal lymphatics without taking advantage of the chylomicron pathways. Du et al. wanted to enhance the potency of parenteral vaccinations to better prime mucosal immunity in the gut [93]. To do so, they loaded all-trans retinoic acid (a*t*RA) and lipid 3β-[*N*-(*N*,*N*-dimethylaminoethane)carbamoyl] cholesterol hydrochloride (DC-Chol) into a poly(lactic-*co*-glycolic acid) (PLGA) nanoparticle coated with CpG oligodeoxynucleotides (CpG) [93]. a*t*RA has been reported to induce C-C chemokine receptor 9 (CCR9) expression that leads to dendritic cell homing to the gut [93]. CDC-Chol was used to better incorporate CpG into the nanoparticle formulation [93]. CpG was chosen as the load as it has been reported to activate the draining LN accumulation of dendritic cells [93]. When the ovalbumin-loaded PLGA lipid nanoparticles (PLNP) were delivered to mice, it was found that ovalbumin internalization was 6-fold higher compared to when only a mixture of ovalbumin-a*t*RA-CpG was delivered [93]. Additionally, the number of antigens residing in the cytoplasm was higher in the PLNP group and there was a significant increase in expression of major histocompatibility complex-I (MHC-I) and activation markers, CD80 and CD86, in dendritic cells [93]. When looking specifically at skin draining lymph nodes and MLN, there was more PLNP-induced antigen primed dendritic cells in the skin draining lymph nodes on day 2 compared to MLNs [93]. However, there was an increase in dendritic cells in MLNs on day 6, indicating a successful gut-homing receptor switch [93]. To further prove this, the group also looked at the migration of T cells and found that T cells also increased from day 2 to 6 [93]. These studies pave the way for future lymph-node-targeting therapeutics by illustrating that lipid nanoparticles can be utilized to target the immune system without requiring the chylomicron pathway.

#### 4.2.2. Non-Lipid-Based Nanomaterials

Previous work has shown that exosomes (EXOs) provide great lymphatic accumulation after intradermal injection and exosomes are primarily transported by lymphatic vessels [94]. Choi et al. used serum-derived 50 nm exosomes to get enhanced transport of their therapeutic to the lymph nodes [95]. They conjugated polyethylene glycol (PEG) and mannose on the surface of the serum-derived exosome (EXO-PEG-man) to inhibit nonspecific binding and to target CD206 receptors on dendritic cells, respectively [95]. Research showed that when DC2.4 cells were treated with EXO-PEG-man, more EXO-PEG-man was internalized compared to control groups [95]. This suggests that these exosomes could be preferentially internalized by CD206-expressing cells [95]. When fluorescent EXO-PEG-man was delivered to the hind paw of mice, fluorescent EXO-PEG-man accumulated more in the popliteal compared to the inguinal lymph node [95]. However, there was no significant difference in lymph node accumulation between fluorescent EXO-PEG-man, fluorescent EXO-PEG-biotin (control), and fluorescent EXO [95]. The researchers also found that all EXO-based formulations significantly increased accumulation compared to free fluorophores [95]. This suggests that EXOs provide an adequate carrier for therapeutic loads to be more readily delivered to the lymph node.

**Figure 2:**
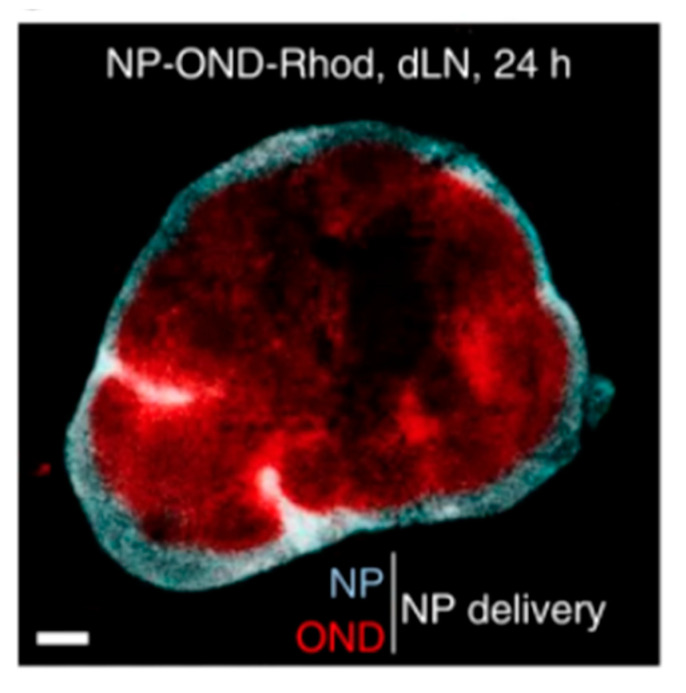
Lymph node cross section containing nanoparticle and OND linkers. Reproduced with permission from [70]. Copyright Springer Nature, 2020.

Once nanomaterials have made their way to the lymph node, only those with molecular weights < 70 kDa can pass through the conduit system to access the adaptive immune cells housed in the lymph node cortex and paracortex [83]. However, to get materials to the lymph node via lymphatics, it is known that nanomaterials must have a size of 10–250 nm to penetrate through the extracellular tissue space and enter lymphatic vessels [83]. Schudel et al. sought to address the discrepancy in size requirements by designing a multistage drug delivery system. They utilized a nanomaterial system in which model therapeutic cargo < 70 kDa was conjugated to poly(propylene sulfide) (PPS) nanoparticles using oxanorbornadiene (OND) linkers that are pH- and solvent-sensitive [96]. The idea was to deliver the nanoparticle system to the lymph node, and once in the lymph node, the OND linkers would be cleaved, freeing the smaller therapeutic load and allowing it to exit the conduit system and enter the lymph node cortex and paracortex [96]. Ten minutes after injection, nanoparticles (27 ± 1 nm in diameter) were found to travel from the injection site to skin draining lymph nodes [96]. To track lymph node biodistribution in vivo, the group labelled the load with cleavable rhodamine and labelled the nanoparticles with non-cleavable Alexa Fluor 647 [96]. After 24 h, Alexa Fluor 647 stayed in the perimeter of the lymph node, while rhodamine was found in the deeper paracortex of the lymph node (Figure 2) [96]. The paracortex is home to B and T cells, which are integral in the formation of the adaptive immune response. Delivering cargo directly to this area will help therapeutics be more potent and will also mean less concentrations of drugs required to elicit a strong immune response.

### 4.3. Immune Cell Targeting

Researchers have made great strides in targeting specific cells for the treatment and prevention of disease. While protecting cargo in drug delivery systems is pivotal in therapeutic efficacy, it does not necessarily improve specificity and potency. There are a number of strategies that researchers are using to target specific cell types: physical, physiological, and biological [97]. In theory, physical targeting of therapeutics to the tissue of interest is the simplest to achieve. However, because most organs are difficult to navigate to, even using specialized equipment, it is not a feasible pathway for most drug systems. Physiological methods are governed by cell concentrations and depend on naturally occurring processes in the body to successfully transport carriers across various barriers [97]. Chylomicron formation is a great example of physiological methods that take advantage of natural pathways to move molecules across the gut barrier. There are three ways in which biological targeting can be achieved: using cell-homing machinery, expressing cell factors that aid in proliferation and survival, and including markers on the surface of cells that have an affinity for a specific target site [97]. Since these methods specifically target cell types, this can potentiate therapeutics and reduce negative systemic side effects. While these strategies can be applied to just about any cell type, they become highly advantageous for targeting immune cells. Immune cells constantly survey the body for foreign materials to create a defensive response against harmful invaders. Targeting immunotherapies to specific immune cells will be helpful to shape an immune response quickly and efficiently against acute and chronic conditions. It may be particularly effective to target migratory immune cells that travel from systemic circulation via peripheral tissues to the lymph nodes, where they initiate and shape the adaptive immune response [98].

Wang et al. used grapefruit-derived nanovesicles (GDNs) to target macrophages in the gut to alleviate inflammation due to colitis [99]. Naringins, compounds naturally found in grapefruit, are hydrolyzed into naringenin by the intestinal microflora and have anti-inflammatory and anticolitic effects [99]. GDNs that were orally administered to mice accumulated in the middle and distal portions of the small intestine, cecum, and colon [99]. The researchers found that GDNs colocalized with F4/80+ macrophages in the gut, including in the Peyer’s patches and MLN. Additionally, they found that GDNs appeared to accumulate in macrophages found in the spleen and liver [99]. The group sought to determine if their GDNs could ameliorate colitis in mice by assessing the production of TNF-α, IL-1β, and IL-10 by macrophages in the inflamed colon. Untreated mice produced 347 ± 37 pg/mL of TNF-α, 150 ± 14 pg/mL of IL-1β, and 315 ± 38 pg/mL of IL-10 [99]. However, GND-treated mice secreted significantly less of the respective TNF-α (222 ± 20 pg/mL) and IL-1β (108 ± 15 pg/mL) [99]. Finally, they loaded methotrexate (MTX), an anti-inflammatory drug, into their GDNs and delivered them to mice with colitis. They found that MTX-loaded GDN successfully targeted F4/80+ macrophages in the lamina propria and reduced colitis-induced body weight loss [99]. This method of reducing local inflammation in the gut can pave the path for future therapeutics by taking advantage of the local immunity found within the gut, which could induce a more potent response, decreasing the need for multiple doses of drug.

RNA therapeutics have recently gotten considerable attention. In colitis, blocking specific cytokines or receptors is thought to be a curative option for patients with colitis, but it is typically insufficient and temporary [100]. Dammes et al. used a lipid nanoparticle formulation to deliver a siRNA target to gut-homing leukocytes as a more permanent treatment solution [100]. They aimed to target the high affinity conformation of α_4_β_7_ by conjugation of one of its ligands, mucosal vascular addressin cell adhesion molecule-1 (MAdCAM-1), to the surface of the lipid nanoparticles via a PEG linker [100]. The group only used binding domains D1 and D2 of MAdCAM-1 to maximize specificity [100]. The IFN-γ gene was used as a therapeutic agent due to its large role in colitis [100]. siIFN-γ loaded NP (D1D2-siIFN-γ LNP) were administered to mice after 4, 6, 8, and 10 postinduction of colitis [100]. IFN-γ levels in the colon decreased by 2.5-fold in the D1D2-siIFN-γ LNP group compared to the control group [100]. IFN-γ also modulates TNF-α expression and NF-ĸB signaling [100]. Therefore, the authors expected a decrease in cytokine secretion with IFN-γ silencing. They found that tissue TNF-α, blood IL-6, and blood IL-1β levels were significantly decreased in D1D2-siIFN-γ LNP-treated mice [100]. The length of the colon in D1D2-siIFN-γ LNP-treated mice was also significantly higher (~7 cm), indicative of an ameliorated colitis, compared to control groups (~4.5 cm) [100].

Targeted delivery to CD8+ effector T cells has also gained popularity in recent years. Schmid et al. modulated immunity by targeting nanoparticles to CD8+ T cells using anti-CD8a F(ab′)_2_ fragments. Between 90% and 100% of the CD8+ T cells were successfully bound to nanoparticles conjugated with anti-CD8a F(ab′)_2_ only 1 h after injection in mice. The group also sought to inhibit CD8+ T cell exhaustion by conjugating anti-PD-1 to the surface of the nanoparticles. Mice were inoculated with tumors and these were allowed to grow to ~400 mm^3^. One hour after nanoparticle injection, immune cells were collected from the tumors, and ~5% of PD-1^+^ cells were bound to nanoparticles. There was also a 10-fold increase in PD-1^+^ cells bound to nanoparticles in the blood. While this application is for tumors, the same concept can be used to target CD8+ T cells in the gut, ameliorating proinflammatory responses against chronic diseases such as colitis or colon cancer.

**Table 2:** Summary of nanoparticles utilized to target the gut, size, mechanism of targeting, and the target cell type/region.

Nanomaterial	Dimension	Mechanism of Targeting	Targeted Cell Type/Region	Sources
Thiol-organosilica nanoparticles	<700 nm	Transcellular and paracellular transport pathways	M cells and CD11+ cells	[70]
Lipid–polymer hybrid nanoparticle	300–400 nm	Mucus sticking	Peyer’s patches	[71,72]
Chitosan nanoparticle	<300 nm	Permeation enhancer	M cells	[73]
Targeting peptide nanoparticle	<250 nm	Adherence to specific M cell sugar residues	M cells	[75,76,77,78,79,80,81,82]
Lipid nanoparticles	<500 nm	Chylomicron formation	Enterocytes; intestinal lymphatics	[85,86,87,88,89,90,91,92,93,94]
Exosomes	50 nm	Receptor targeting	Targeting receptors on dendritic cells (i.e., CD206)	[95]
Poly(propylene sulfide) nanoparticles	<250 nm	Cleaving of linkers	Cortex and paracortex of lymph node	[96]

### 4.4. Taking Advantage of Oral Tolerance

Oral tolerance is one of the body’s ways to prevent immune responses against non-harmful foreign materials such as food and commensal bacteria (microbiome). New research has shed light on some site-specific mechanisms of oral tolerance, such as a regional dependency on lymph nodes in the earlier small intestine that process materials drained from duodenal and jejunal sections [43]. This naturally occurring mechanism has received a significant amount of attention as a therapeutic target, as it provides an “easy” way to deal with diseases where the immune response has gone awry. Several studies have been conducted using nanomaterials to take advantage of oral tolerance, through targeting either local immunity or lymph nodes, as well as indirectly utilizing the existing oral tolerance mechanisms for autoimmune or allergic diseases.

Oral tolerance has been utilized to enhance therapeutics re-establishing tolerance against autoantigens in autoimmune diseases. Several studies have focused on treating or preventing diabetes and arthritis. Neumann et al. demonstrated that delivery of glucagon receptor (Gcgr)-siRNA delivered in lipid nanoparticles reduced blood glucose levels in STX diabetic mice for 3 weeks. They found that their lipid nanoparticles improved glucose tolerance, and normalized plasma ketones levels, while leptin therapy normalized blood glucose levels, oral glucose tolerance, and plasma ketones, and suppressed lipid metabolism [101]. In high-fat diet STZ diabetic mice, however, leptin therapy showed no beneficial effects while the siRNA-loaded formulation lowered blood glucose levels and improved oral glucose tolerance for two months. Xu et al. also used lipid nanoparticles to deliver glucagon-like peptide-1 to the gut and demonstrated that their nanoparticle system enhanced GLP1 production in vitro and in vivo and subsequently improved glucose tolerance and insulin resistance [102]. Furthermore, chronic treatment reduced diet-induced obesity, fat mass, hepatic steatosis, and infiltration and recruitment of immune cells into affected tissues. Chen et al. reported that using dual targeting to deliver heat shock protein 6 to gastrointestinal dendritic cells, which are known to induce antigen specific tolerance, induced antigen specific tolerance and prevented mice from developing diabetes [103]. The nanoparticle system contained both M cell-targeting RGD motifs as well as mannose to target dendritic cells. This nanoparticle system increased uptake of heat shock protein in Peyer’s patches 3-fold higher than the free solution, and the formulation induced more regulatory T cells and a switch from a type 1 to a type 2 immune response, which they hypothesized was responsible for the more effective prevention of diabetes. Work by Lee and Kim et al. has also demonstrated that collagen-induced arthritis can be prevented by immunizing mice with nanoparticles containing type II collagen, taking advantage of the body’s natural oral tolerance mechanisms indirectly [104,105]. The tolerogenic effect can be further enhanced by delivering dexamethasone, an anti-inflammatory drug, along with an antigen orally [106]. Kim et al. demonstrated that dexamethasone-loaded nanoparticles also containing the model antigen ovalbumin reduced the number of cytotoxic T cells and increased the number of regulatory T cells specific to ovalbumin. This treatment also reduced ovalbumin specific antibodies. These data demonstrate that oral tolerance mechanisms can be indirectly targeted using nanomaterials to reduce immune responses against autoantigens and this can be further enhanced using anti-inflammatory drugs.

Researchers have also taken advantage of oral tolerance to treat allergies, including food allergies. Oral allergen immunotherapy may cause severe anaphylactic reactions, and nanomaterials offer protection from this by shielding the allergens from antibodies and immune cells by encapsulating them inside a shell of polymer or lipid. Several research groups have devised strategies to encapsulate peanut protein into small nanoparticles to both deliver the protein to intestinal immune cells and shield it from antibodies and other rapid immune mechanisms that could cause severe allergic responses. Reboucas et al. used spray-dried or lyophilized polyanhydride nanoparticles loaded with peanut proteins that were administered orally [107]. They demonstrated that their nanoparticle system reduced the allergic type 2 cytokines (IL-4 and 5) in the spleen while increasing anti-allergic type 1 (IFNγ) and regulatory (IL-10) cytokines. The group then demonstrated that their nanoparticles diffused through pig intestinal mucus, thus ensuring the formulation effectively reaches the biggest surface area in the gut. They found that mice receiving three doses of encapsulated peanut protein had increased survival and lower levels of mast cell proteases after a peanut challenge [108]. The same group also developed a nanoparticle system that can target the gut Peyer’s patches by attaching Gantrez to the polyanhydride nanoparticles and showed that oral immunization with three doses of nanoparticles reduced IgE levels and protected mice against severe anaphylaxis [109]. Srinivasta et al. demonstrated that PLGA nanoparticles containing the proinflammatory TLR 9 agonist CpG and peanut extract could protect mice from anaphylaxis [110]. Their nanoparticle treatment reduced symptom scores, antihistamines, and change in body temperature, after just one treatment of PLGA nanoparticles followed by five oral challenges with peanut extract. Despite prior sensitization to peanut extract, the nanoparticles did not cause anaphylaxis and in fact reduced peanut-specific IgE and IgG1, while increasing peanut-specific IgG2a, which is known to inhibit allergic responses. Additionally, they found that splenocytes had reduced IL-4, 5, and 13 production and increased IFNγ production after ex vivo restimulation with peanut extract. Altogether these data demonstrate the potential for using nanomaterials to take advantage of oral tolerance mechanisms for treating allergic diseases, which could have a huge impact on the globally growing number of people suffering from allergies.

### 4.5. Oral Vaccines

**Figure 3:**
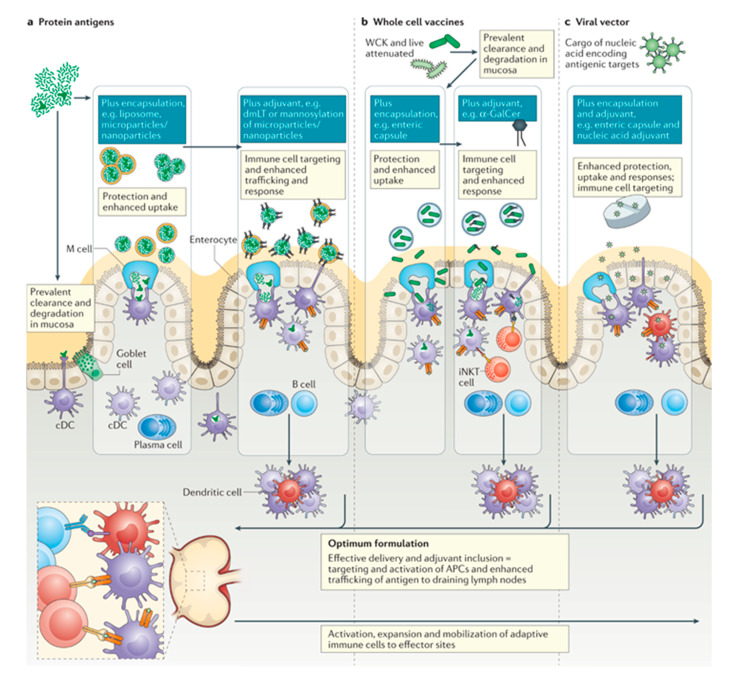
Summary of three different types of oral vaccines and their mechanisms of entry into the gut. These mechanisms are not limited to vaccine delivery, but are also used to deliver other drug delivery systems. Reproduced with permission from [88]. Copyright Springer Nature, 2021.

Vaccines are one of our most powerful tools in reducing the burden of infectious diseases worldwide, providing an effective strategy to provide protection to vaccinated individuals as well as the population at large. Despite this, the global morbidity due to infectious disease is still incredibly high [111,112]. Recently, the severe acute respiratory syndrome coronavirus 2 (SARS-CoV-2) pandemic has demonstrated the persistent threat mucosal diseases pose, and the continual need for the development of effective mucosal vaccines. Additionally, enteric pathogens causing diarrhea disease are the 8th leading cause of morbidity worldwide, posing an increased risk to children and those living in lower-income countries and communities. With community protection reliant on prevention of colonization of pathogens within the gut and the prevention of low-grade infection, cost-effective oral vaccines targeting the mucosa are a key tool to combat these prevalent diseases [113]. Aside from providing a potentially cheaper, and more available alternative to parenteral vaccines, oral and mucosal vaccines can increase efficacy by inducing strong mucosal cellular and humoral immune responses that may be able to induce sterilizing immunity. With advances in adjuvant, antigen, and formulation development and discovery, vaccines are being engineered to have higher pathogen specificity and narrower immunogenicity. However, these advances have been slow to translate to oral vaccines. As of now, there are nine mucosal vaccines licensed for use (eight oral, one nasal), all relying on whole or attenuated microbial components, likely due to the higher tolerance observed with the oral delivery route, and the general susceptibility to degradation of materials delivered orally (Figure 3) [114,115]. Most successful oral and mucosal vaccines are able to illicit responses from key immune cells, including antigen-presenting cells and other populations of cells enriched within the mucosa, including innate lymphoid cells, mucosal activated invariant T cells, natural killer cells, and γδ T cells (Figure 3) [116,117]. We would like to point the readers to several excellent recent reviews on bio- and nanomaterials for oral vaccines [82,118,119].

### 4.6. Outlook

The described studies in this review article demonstrate the vast applications for targeting gut immunity, ranging from therapeutic treatments of local diseases to vaccines and improved delivery of therapeutics. To translate these therapies to the clinic, patient compliance is a major hurdle to overcome. However, the GI tract’s ease of accessibility and potential for at home treatments can help alleviate this hurdle, as oral therapeutics are easy to administer and commonly used already. Most therapeutic strategies in development have focused on delivering materials to either mucosal immune sites such as Peyer’s patches in the gut, or to take advantage of oral tolerance mechanisms. A major underexplored area is targeting the gut lymph nodes that could provide systemic immunity against pathogens in a form easier to translate to patients via oral delivery (instead of injection). Only few oral vaccine strategies have been translated to the clinic, and many have been found unsuccessful once tested in human trials. Oral vaccine efficacy could be enhanced by providing more effective strategies to target and activate mucosal immunity both locally and in the lymph nodes. Overall, the field has made major progress, but there is still room for growth and improvement in targeting gut immunity for therapeutic applications.

### 4.7. Literature Search Method

The authors used literature found mainly through PubMed to identify appropriate articles for this review. Keywords included, but were not limited to, the following: GALT, oral vaccine, lipid, nanoparticles, exosomes, lymphatic, lymph nodes, peptide, mucus, tolerance, gastrointestinal, chylomicron, etc. Criteria of inclusion and exclusion were at the discretion of the authors. Generally, articles that most accurately described the needed subject were included.

## Figures and Tables

**Table 1:** Schematic representation of the structure of nanoparticles and their advantages and disadvantages.

	Advantages	Disadvantages	GI Application	Sources
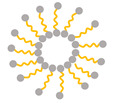 **Liposomes**	Shields encapsulated drugs Nontoxic Biodegradable	Low solubility Short circulation time Very sensitive to environmental changes	Liposomes can be formulated to release poorly soluble agents in GI regions of interest. Liposomes are useful to encapsulate poorly soluble drugs.	[44,45,46,47,48,49,50,51]
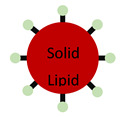 **Solid Lipid Nanoparticles**	Biocompatible Flexible formulation can allow tissue specificity	Difficulty scaling production	Attachment of homing molecules (i.e., bile salts) have been used to enhance uptake in GI tissues. Solid lipid nanoparticles are useful to encapsulate poorly soluble drugs.	[52,53,54,55,56,57,58]
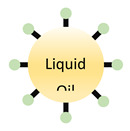 **Nanoemulsion**	Generally formulated with GRAS material Can dissolve large quantities of low solubility drugs	Very sensitive to environmental changes	Nanoemulsions can greatly improve solubility of both hydrophilic and hydrophobic agents.	[59,60]
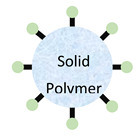 **Polymeric Nanoparticle**	Biodegradable Nontoxic Flexible formulation can allow tissue specificity	Difficult scaling production	Attachment of homing molecules, including bile salts and vitamin B12, have been used to enhance uptake in GI tissues.	[61,62,63,64,65,66,67,68,69]

## Data Availability

Not Applicable.
